# The Toxic Influence of Excess Free Iron on Red Blood Cells in the Biophysical Experiment: An In Vitro Study

**DOI:** 10.1155/2022/7113958

**Published:** 2022-02-26

**Authors:** E. Kozlova, E. Sherstyukova, V. Sergunova, A. Kozlov, O. Gudkova, V. Inozemtsev, A. Chernysh

**Affiliations:** ^1^Federal Research and Clinical Center of Intensive Care Medicine and Rehabilitology, V.A. Negovsky Research Institute of General Reanimatology, Moscow, Russia; ^2^Sechenov First Moscow State Medical University (Sechenov University), Moscow, Russia; ^3^Federal State Budget Educational Institution of Higher Education, M. V. Lomonosov Moscow State University (Lomonosov MSU), Faculty of Physics, Moscow, Russia

## Abstract

Iron is needed for life-essential processes, but free iron overload causes dangerous clinical consequences. The study of the role of red blood cells (RBCs) in the influence of excess free iron in the blood on the pathological consequences in an organism is relevant. Here, in a direct biophysical experiment *in vitro*, we studied the action of free iron overload on the packed red blood cell (pRBC) characteristics. In experiments, we incubated pRBCs with the ferrous sulfate solution (Fe^2+^). Wе used free iron in a wide range of concentrations. High Fe^2+^ concentrations made us possible to establish the pattern of the toxic effect of excess iron on pRBCs during a reduced incubation time in a biophysical experiment *in vitro*. It was found that excess free iron causes changes in pRBC morphology, the appearance of bridges between cells, and the formation of clots, increasing the membrane stiffness and methemoglobin concentration. We created a kinetic model of changes in the hemoglobin derivatives. The complex of simultaneous distortions of pRBCs established in our experiments can be taken into account when studying the mechanism of the toxic influence of excess free iron in the blood on pathological changes in an organism.

## 1. Introduction

Iron is a significant metal for various processes in biological systems, but there is no active mechanism for the excretion of excess iron from the body under conditions of iron overload (hemochromatosis) [1–3].

Overload of free iron in the blood has different origins: chronic iron overload is a common complication of repeated packed red blood cell (pRBC) transfusions [1, 4–6]; disorders of iron metabolism and chronic iron overload are observed in a number of acute and chronic inflammatory diseases and in acute respiratory distress syndrome [7, 8]; cell-free hemoglobin can become a source of excess free iron, arising under the action of hemolytic poisons [9], sickle cell anemia [10], hemodialysis [11], cardiac bypass [12], pulmonary arterial hypertension [13, 14], and sepsis [15–18], with phagocytosis of damaged RBCs [19, 20], hereditary hemochromatosis [21], and viral diseases [22–25], including SARS-CoV-2 [8, 26, 27]. Serious toxic effects were observed in a number of extreme cases [28–30].

Excess iron accumulation causes organ dysfunction [2, 4, 8, 31–36]. Iron overload is especially dangerous for critically ill patients [37–39]. Often, pathological consequences are associated with the action of various factors on elements and processes in the blood in the cardiovascular system: the coagulation system is changed, blood clotting, hemostasis, and thrombosis that is clinically significant are arisen [31, 40, 41].

In these cases, RBCs are the key elements. Therefore, we raised a question what is happening or can potentially happen in RBCs under toxic influence of free blood iron. The changes in RBC morphology after influence of iron on them are shown in a number of scientific articles. There are studies that have established a change in the morphology of RBCs *in vitro* as a result of the action of free iron ions in high concentrations. The shape change was observed in the blood of people with hemochromatosis [36, 42].

As a rule, RBC shape distortion and an increase in their membrane stiffness are associated with the acceleration of oxidative processes under the action of physical and chemical factors [43–46].

Free iron is potentially extremely toxic due to its ability to generate reactive oxygen species (ROS), which are involved in various redox processes [9, 31, 47–49]. One of the consequences and at the same time a biomarker of the development of oxidative processes in the blood is the formation of methemoglobin (MetHb) [50, 51].

In this work, we conducted direct biophysical experiments to solve the problem of revealing and quantitative estimation of toxic effects of free iron on pRBCs in an *in vitro* model. Wе used free iron in a wide range of concentrations. High Fe^2+^ concentrations made us possible to establish the pattern of the toxic effect of excess iron on pRBCs during a reduced incubation time in a biophysical experiment *in vitro*. For quantitative registration of pRBC modifications, we used atomic force microscopy (AFM). Atomic force spectroscopy (AFS) allowed us to measure the local Young's modulus of native membranes. High-resolution digital spectroscopy and the nonlinear curve fitting method for the analysis of optical spectra allowed us to experimentally establish the kinetics of MetHb formation under the influence of excess Fe^2+^. We created a mathematical kinetic model of changes in the concentrations of Fe^2+^ and hemoglobin derivative concentrations, which adequately describes the experimental data and allows to estimate the rate constants of redox processes.

## 2. Materials and Methods

### 2.1. Blood Products

Packed red blood cells were obtained from the clinical centers of blood transfusion in Moscow, Russian Federation. All of the experiments were conducted in accordance with guidelines and regulations of the Federal Research and Clinical Center of Intensive Care Medicine and Rehabilitology, V.A. Negovsky Scientific Research Institute of General Reanimatology, Moscow, Russian Federation. All of the experimental protocols were approved by this institute.

A total of 5 bags with pRBCs were used in the study. pRBC units were prepared from the whole blood (450 ml ± 10%) by removing the plasma fraction after centrifugation. Leukocyte-depleted pRBCs in airtight bags were stored with standard CPD anticoagulant (63 ml) (citrate, phosphate, and dextrose) and SAGM additive solution (100 ml) (saline, adenine, glucose, and mannitol) at a temperature of +4°С. The hematocrit of the pRBCs was 60–65%. Samples of pRBCs for *in vitro* experiments were withdrawn on days 3–5.

**Figure 1:**
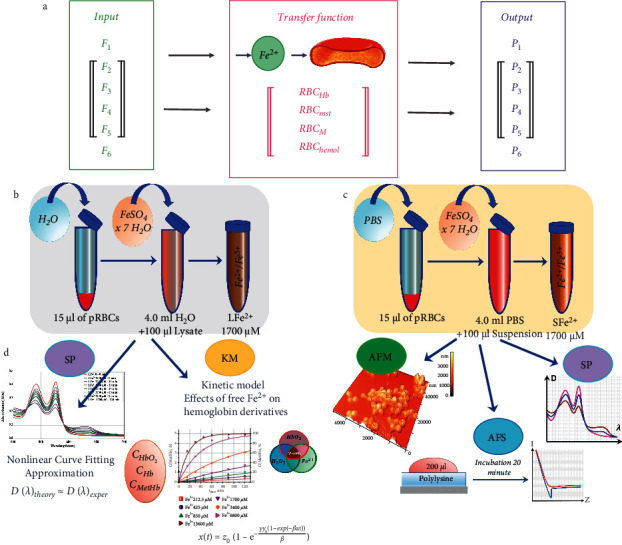
RBCs as a transfer link in the influence of factors determining an excess of Fe^2+^ on clinical consequences in organisms. (a) A schematic representation of the influence of various factors determining excess Fe^2+^ on clinical consequences in the organism. Input includes *F*_*i*_: factors determining excess Fe^2+^; transfer function includes pathological changes in RBCs: RBC_Hb_, content of hemoglobin derivatives; RBC_mst_, membrane stiffness; RBC_*M*_, cell morphology; RBC_hemol_, hemolysis; output includes *P*_*i*_: pathological clinical consequences. (b) Experimental design for pRBC lysate. The designation of pRBC lysate with iron is LFe^2+^ 1700. (c) Experimental design for pRBC suspension. The designation of pRBC suspension with iron is SFe^2+^ 1700. (d) Methods to study the action of excess Fe^2+^ on RBCs. AFM, atomic force microscopy; AFS, atomic force spectroscopy; KM, kinetic model; SP, spectrophotometry.

Stages of the study are shown in detail in Figure 1.

### 2.2. Preparation of Ferrous Sulfate Solution

A ferrous sulfate stock solution (solution A) was prepared by dissolving 20 mg iron (II) sulfate heptahydrate (FeSO_4_·7H_2_O_2_) (Sigma-Aldrich, USA) with 1.0ml of PBS (pH 7.4)/distilled water, *C*_Fe^2+^_ = 71.9 mM. Then, 100 *μ*l of solution A were added to 4 ml of distilled water/PBS (solution B). The initial concentration of Fe^2+^ in lysate/suspension was 1700 *μ*M, and this is designated as Fe^2+^1700. To study the influence of Fe^2+^ in initial various concentrations, we changed solution A to receive *C*_Fe^2+^_ from 212.5 to 13600 *μ*M. Wе used free iron in a wide range of concentrations *C*_Fe^2+^_.

High Fe^2+^ concentrations make it possible to establish the pattern of the toxic effect of excess iron on pRBCs during a reduced incubation time in a biophysical experiment *in vitro*.

### 2.3. pRBC Lysate and Suspension Preparation *In Vitro*

To prepare the lysate and suspension of pRBCs *in vitro*, the components of the solution in which the pRBCs were stored were removed. To do this, 200 *μ*l of pRBCs was washed in 1 ml of phosphate buffer saline (PBS) at pH 7.4 (MP Biomedicals, France). This suspension was centrifuged at 2,000 rpm for 5 minutes in a Universal 320 centrifuge (Andreas Hettich GmbH & Co. KG, Germany). This procedure was carried out twice. Then, the lysate (L) was prepared by mixing 15 *μ*l of pRBCs and 100 *μ*l of distilled water, and the pRBC suspension (S) was prepared by mixing 15 *μ*l of pRBCs and 100 *μ*l of PBS. Then, 100 *μ*l of lysate/suspension were diluted in 4.1 ml of solution B. The time of iron interaction with pRBCs was named as the incubation time and designated as *t*_inc_. Stages of the experiment are shown in Figures 1(b) and 1(c).

### 2.4. Spectrophotometry

For the spectrophotometry study, 100 *μ*l of lysate was diluted in 4 ml of distilled water, or 100 *μ*l of suspension was diluted in 4 ml of PBS with 100 *μ*l solution A as shown in Figures 1(b) and 1(c). The working solution with ferrous sulfate and lysate was designated as LFe^2+^ 1700, and the similar suspension solution was designated as SFe^2+^1700; the solution without ferrous sulfate for lysate was designated as LFe^2+^ 0, and the suspension was designated as SFe^2+^ 0. The incubation times of the lysate of pRBCs were 0, 15, 30, 45, 60, 90, 120 min, and 24 hours, and for the suspension of pRBCs, the incubation times were 1, 5, and 24 hours.

To determine the concentration of hemoglobin derivatives, the optical absorption spectra were measured using a Unico 2800 digital spectrophotometer (United Products & Instruments, USA). The experimental spectrum *D*(*λ*)_exp_ was measured in the wavelength range of 500–700 nm with steps of 0.5 nm. The concentrations of hemoglobin derivatives (HbO_2_, Hb,  and MetHb) were measured according to their individual absorptivities at different wavelengths using the nonlinear curve fitting method of experimental spectra.

### 2.5. Nonlinear Curve Fitting Optical Spectra

For the determination of hemoglobin derivatives, the nonlinear curve fitting method was used as described in [52, 53]. Briefly, it is necessary to experimentally obtain the set of optical densities measured at corresponding wavelengths *D*_*l*_(*λ*_*l*_)_exper_, where *l* is the number of wavelengths and *λ*_*l*_ is the set of wavelengths. Then, we define our own function *D*_*l*_(*λ*_*l*_)_theor_ by using Origin's flexible Fitting Function Builder by Origin Pro 2019 (OriginLab Corporation, USA):

(1) $$\begin{array}{c} D_{l}{\left(\lambda_{l}\right)}_{\text{theor}}=\;\varepsilon_{{\text{HbO}}_{2,l}}C_{{\text{HbO}}_{2}}L+\varepsilon_{\text{Hb,}l}C_{\text{Hb}}L+\varepsilon_{\text{MetHb},l}C_{\text{MetHb}}L+K+\frac{S}{\lambda_{l}^{4}}. \end{array}$$

In this equation, there are known values: molar absorptivity coefficients at given wavelengths *λ*_*l*_ (*ε*_HbO_2,*l*__, *ε*_Hb,*l*_, and *ε*_MetHb,*l*_) [50] and the thickness of the layer *L*. There are also unknown values: concentrations of corresponding hemoglobin derivatives (*C*_HbO_2__, *C*_Hb_, and *C*_MetHb_) and coefficients of scattering (*K* and *S*). These parameters must be determined by fitting the model.

This function is created according to biophysical considerations. Because red blood cells are studied in PBS, it is necessary to consider not only the absorption but also the scattering processes. The intensity of these processes will be different for various wavelengths.

The coefficient *K* describes the scattering of light on pRBCs when the wavelength is smaller than the diameter of pRBCs, *λ* ≪ *d*. The coefficient *S* corresponds to Rayleigh scattering when the scattering particles are very small, *λ* ≫ *d*′. In the nonlinear curve fitting model, the experimental optical density data (*D*_*l*_(*λ*_*l*_)_exper_) are used instead of *D*_*l*_(*λ*_*l*_)_theor_. Computing the fitted values in nonlinear regression is an iterative procedure performed using the Levenberg–Marquardt algorithm. Iteration to adjust parameter values continues to make data points closer to the theoretical curve. The adjusted R-squared is the measure of the goodness of fit.

The concentration of total hemoglobin *C*_total_ is the sum of hemoglobin derivative concentrations, in *mmol/l*. The percentage of each derivative in pRBCs is the ratio, particularly,

(2) $$\begin{array}{c} C_{\text{MetHb}}\%\;=100\frac{C_{\text{MetHb}}}{C_{\text{total}}}\%. \end{array}$$

### 2.6. Kinetic Model of Change of Hemoglobin Derivative Concentrations under the Influence of Fe^2+^

The mathematical modeling was used to study the dynamics of the increase in the concentration of MetHb under the actions of ferrous sulfate solution in different concentrations. The kinetics of the Fe^2+^ and H_2_O_2_ interaction as well as the kinetics of an increase in the concentration of MetHb are analyzed. Based on the law of mass action, ordinary differential equations describing the kinetics of chemical reactions were recorded. The kinetic equations were solved using the method of separation of variables, taking into account the initial conditions. Details of the model are given in the text.

The obtained theoretical dependence *C*_MetHb theor_(*t*) was used as the basis for the approximation of the experimental data *C*_MetHb exper_(*t*). As a result, the unknown parameters of the approximation were estimated—the rate constants of the reactions and the concentration of H_2_O_2_.

### 2.7. Atomic Force Microscopy

To obtain images of cells, a smear of pRBC was formed. First, 4.2 ml of pRBC suspension (SFe^2+^0 and SFe^2+^1700) was sedimented by centrifugation at 2,000 rpm for 5 minutes. Next, 50 *μ*l of 1% glutaraldehyde solution (Panreac Quimica S.L.U., Spain) was added to 50 *μ*l of sediment for 4 minutes. To wash the cells, 500 *μ*l of distilled water was added to the pRBC suspension. After centrifugation at 2,000 rpm for 5 minutes, the supernatant was removed and 300 *μ*l of distilled water was added. Then, the sample was centrifuged again, and the supernatant was removed. The smear of sediment was prepared using V-sampler (Vision, Austria).

For the analysis of cell morphology, an NTEGRA Prima atomic force microscope (NT-MDT Spectrum Instruments, Russian Federation) was used. Scanning was carried out in semicontact mode with the NSG01 probe (probe radius 10 nm, resonance frequency 87–230 kHz, and force constant 1–15 N/m) (TipsNano, Estonia). During scanning, fields with sizes of 50 × 50 *μ*m^2^, 30 × 30 *μ*m^2^, and 10 × 10 *μ*m^2^ were selected. The number of dots per line was 1024. The characteristic incubation times for pRBC samples were 1, 5, and 24 hours. Various concentrations of ferrous sulfate were studied. For each incubation time and concentration of ferrous sulfate, images and their profiles were obtained, which made it possible to estimate the spatial characteristics: *L* is the spatial period between the minima of the structure, and *h* is the average height from the concaves to the maxima. The SPM Nova software (NT-MDT Spectrum Instruments, Russian Federation) was used to record AFM images.

### 2.8. Atomic Force Spectroscopy

The stiffness of pRBC membranes is characterized by Young's modulus (E). The method of sedimentation was used to prepare the sample. To do this, 300 *μ*l of pRBC suspension (SFe^2+^1700 and SFe^2+^0) was applied to glass coverslips coated in poly-L-lysine hydrobromide (MP Biomedicals, France) and was left for 30 minutes for adhesion. Then, the coverslips were washed in PBS. For this, the type of cantilever SD-R150-T3L450B-10 (Nanosensors, Switzerland) was used (probe radius 150 nm, resonance frequency 21 kHz, force constant 1 N/m). Force curves were measured only on native cells, without adding chemical fixatives. Measurements were carried out after 1 and 24 hours.

### 2.9. Statistical Analysis

For each bag, 3 smears of pRBCs were prepared. On each smear, 3 fields of 50 × 50 *μ*m^2^ were scanned. For one bag, morphology analysis was carried out for an average of 500 cells. In total, approximately 2500 cells were studied. For each bag, 100 force curves were measured for one sample. A total of 2000 pRBCs were analyzed in the study. For each blood sample for spectrophotometry, the experiments were performed three times.

Statistical processing was performed using the Origin Pro 2019 software. All data are presented as the mean ± SD, and replicate information is indicated in the figure legends. The normality Shapiro–Wilk test for all data was used. The one-way ANOVA test followed by the Tukey's post hoc test for experimental data comparison was used. Pearson correlation analyses were performed to determine the close relationships between MetHb level, hemolysis level, and the average number of discocytes on a smear.

## 3. Results and Discussion

### 3.1. RBCs as a Possible Key Link in the Adverse Effect of Excess Free Iron

Various primary factors *F*_*i*_ (frequent blood transfusion *F*_1_, hereditary hemochromatosis *F*_2_, severe blood loss *F*_3_, bacteria and viruses *F*_4_, sepsis *F*_5_, and chemical pharmaceuticals *F*_6_) results in free iron overload (Fe^2+^) in the blood leading to pathological consequences in the organism *P*_*i*_ (organ dysfunction *P*_1_, atherosclerosis *P*_2_, microvascular pulmonary thrombovasculitis obliterans (in particular, with СOVID-19) *P*_3_, carcinogenesis *P*_4_, intravascular hemolysis *P*_5_, and diabetes *P*_6_). In the chain of these events, RBCs play a key role as the transfer function between the primary factors and the pathological consequences *P*_*i*_, *F*_*i*_ ⟶ RBCs ⟶ *P*_*i*_ (Figure 1(a)).

In this study, we consider the potential distortions of excess Fe^2*+*^ action on RBCs, shown in red in Figure 1(a). In particular, changes in the content of hemoglobin derivatives (RBC_Hb_), and changes in cell morphology (RBC_*M*_), hemolysis (RBC_hemol_), and membrane stiffness (RBC_mst_), arising simultaneously under the influence of excess Fe^2+^ are considered.

We investigated the impact of free Fe^2+^ on the pRBC suspension and lysate (Figure 1(b) and 1(c)) *in vitro* and identified disruption in cell elements. In the experiments, dissolved FeSO_4_ was added to the test sample (Figure 1(b) and 1(c)). Analysis was performed using digital spectrophotometry (Δ*λ* = 0.5 nm), AFM (resolution limit of 0.1 nm in height and 10 nm in length), AFS, and mathematical modeling (Figure 1(d)). More details about the *in vitro* systems are indicated in Materials and Methods.

### 3.2. Nonlinear Kinetics of MetHb Formation Was Registered with Excessive Content of Free Fe^2+^

**Figure 2:**
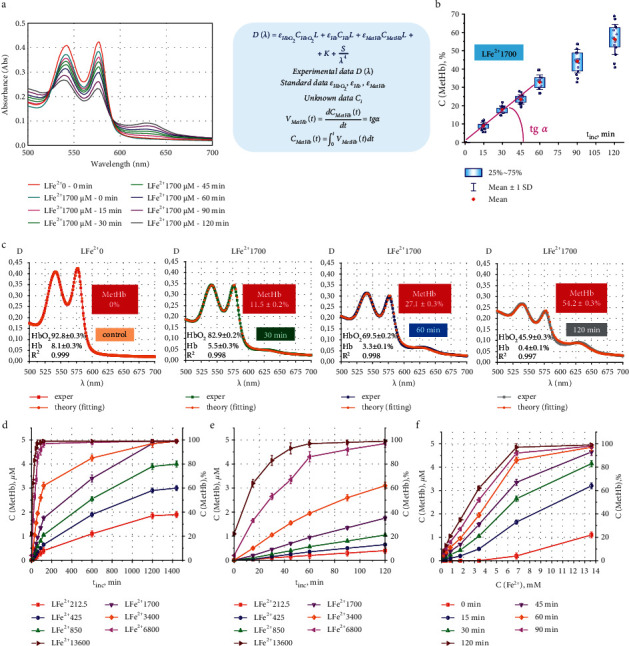
Increasing MetHb content with excessive content of free Fe^2+^. (a) Optical spectra of the LFe^2+^ 1700 sample, incubation time t_inc_ = 0, 15, 30, 45, 60, 90, and 120 minutes. On the right are the equations for calculating the concentrations of hemoglobin derivatives. (b) Increase in *C*_MetHb_(*t*) depending on the incubation time for the LFe^2+^ 1700 sample. Data are presented as box chart, from the 25th to the 75th percentile, whiskers are presented as mean ± SD, and the red square denotes mean. Dots are the experimental data. (c) Fitting results of the experimental data for the LFe^2+^ 0 and LFe^2+^ 1700 samples (t_inc_ = 0, 30, 60, and 120 min). Concentrations of hemoglobin derivatives are shown on each graph and were calculated by the nonlinear curve fitting method. Additionally, the R-square parameter is shown. (d, e) Dynamics of changes in the *C*_MetHb_(*t*) for different initial concentrations of free Fe^2+^ in dependence of time (0–1500 min (d) and 0–120 min (e)); on the graph axes, MetHb concentrations are in µM from the left and in % from the right. Initial concentrations of free Fe^2+^ are shown in different colors. (f) Increase in the *C*_MetHb_(*t*) as a function of the initial free Fe^2+^ concentration. Different colors correspond to different incubation times.

The absorption spectrum of pRBC lysates after the addition of FeSO_4_ salt solution changes significantly over time (Figure 2(a)). When the pRBC lysate is incubated with free iron, the absorption peak at *λ*_3_ = 630 nm appears and grows, so the process of conversion of oxyhemoglobin HbO_2_ to MetHb develops. For Fe^2+^ of 1700 *μ*M during a 60 min incubation, the MetHb content developed almost linearly (Figure 2(b)). After 60 minutes, nonlinear kinetics of the MetHb increase in the pRBC lysate was observed (Figure 2(b)). The instantaneous rate of MetHb formation can be calculated as *V*_MetHb_(*t*)=(d*C*_MetHb_(*t*)/d*t*)=*tgα*; accordingly, by time *t,* its content will be *C*_MetHb_(*t*)=∫_0_^*t*^*V*_*MetHb*_(*t*)d*t*.

Three hemoglobin derivatives play important roles in the absorption process: HbO_2_, Hb, and MetHb. The nonlinear curve fitting method was used to estimate unknown concentrations of the hemoglobin derivatives *C*_HbO_2__, *C*_Hb_, and *C*_MetHb_, which best described the experimental data (Figure 2(a)). For this, the optical spectrum *D*_*l*_(*λ*_*l*_)_exper_ was approximated by the theoretical curve *D*_*l*_(*λ*_*l*_)_theor_, according to which for each wavelength *λ*_*l*_ optical density is a sum of products of the molar absorption coefficient (*ε*_HbO_2,*l*__, *ε*_Hb,*l*_,  and *ε*_MetHb,*l*_) and concentrations *C*_*i*_ for the corresponding hemoglobin derivatives (Equation (1)). Equation (1) was used earlier in our studies [52–54].

Figure 2(c) shows the measured spectra *D*_*l*_(*λ*_*l*_)_exper_ and fitting curves *D*_*l*_(*λ*_*l*_)_theor_, plotted for the parameter *C*_*i*_, calculated by equation (1), to determine the effect of Fe^2+^ 1700 *μ*M on the pRBC lysate with incubation times of 0, 30, 60, and 120 min. For the pRBC lysate, the calculated scattering coefficients *K* and *S* (equation (1)) equaled zero.

Free Fe^2+^ exposure at different concentrations *C*_Fe^2+^_ led to increasing of *C*_MetHb_(*t*). Moreover, the rate of *C*_MetHb_(*t*) growth increased with the increment of the initial concentration of Fe^2^ (Figure 2(d)). Experimental data for incubation times up to 120 min are shown a larger scale in Figure 2(e). In the first 15 min, the initial rates of MetHb formation were *V*_MetHb_(*t*)=*tgα*=0.04%, 0.13%, 0.27%, and 2.2% for Fe^2+^ 212.5, 850, 1700, and 6800 *μ*M, correspondingly.

The steady state level of the *C*_MetHb stst_ and the time to reach it *t*_MetHb stst_ (Figures 2(d)–2(f)) depended on the concentration *C*_Fe^2+^_. Therefore, with *C*_Fe^2+^_ = 6800–13600 *μ*M, *C*_MetHb stst_=97 − 100% and *t*_MetHb stst_=100 − 200min. At lower concentrations, *C*_Fe^2+^_ = 212.5–425 *μ*M, *C*_MetHb stst_=38 − 60%, and *t*_MetHb stst_ > 1200 − 1400min. Up to Fe^2+^ 6800 *μ*M, the dependence of *C*_MetHb_(*C*_Fe^2+^ _) was linear for pRBC lysate incubation times up to 60 min (Figure 2(f)).

**Figure 3:**
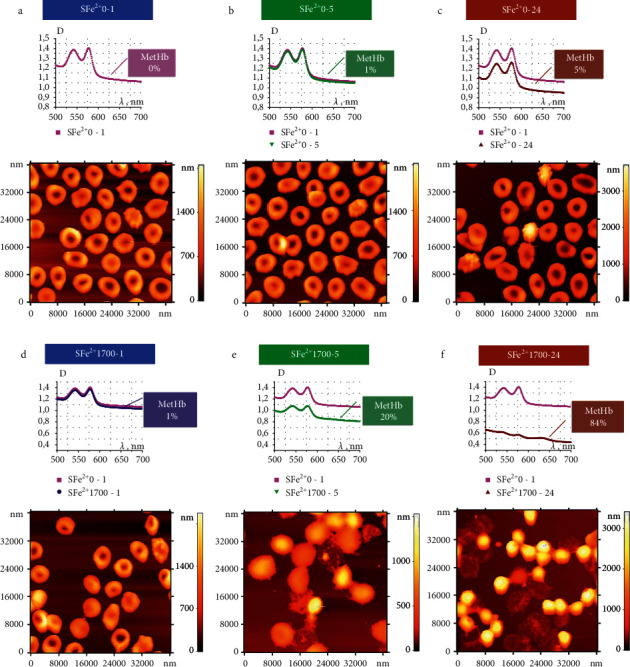
Effects of free Fe^2+^ on the increase in MetHb content and the increase in polymorphisms. (a–c) Optical spectra and AFM images for the pRBC suspension (SFe^2+^ 0), scale 40 × 40 *µ*m^2^. Incubation times were 1 h (a), 5 h (b), and 24 h (c). Color scales of heights are shown for the images. The *C*_MetHb_(*t*) is indicated on each graph. (d–f) Optical spectra and AFM images for the pRBC suspension after exposure to Fe^2+^ (SFe^2+^ 1700), scale 40 × 40 *µ*m^2^. Incubation times were 1 h (d), 5 h (e), and 24 h (f). The *C*_MetHb_(*t*) is indicated on each graph. Color scales of heights are shown for the images.

The formation of MetHb in cells under the influence of Fe^2+^ on the pRBC suspension occurred 5–10 times slower than in the lysate (Figures 2 and 3).

### 3.3. Excess Free Iron Results in pRBC Polymorphism

The result of the interaction of Fe^2+^ and H_2_O_2_ is the formation of a highly reactive radical OH^·^, which attacks lipids and proteins in RBC membranes. It is known that, as a result of the violation of ionic equilibrium during the development of oxidative processes under the action of various physical and chemical factors, cell cytoskeleton and membrane nanosurface changes [46, 55–58], resulting in RBC morphology alterations [45, 56, 58, 59].

In this study, we investigated how MetHb formation was accompanied by changes in pRBC morphology under Fe^2+^ influence (Figure 3). AFM images of typical fragments (40 × 40 *μ*m^2^) of conventional RBC smears are shown in Figure 3. For comparison, controls (pRBC shapes and spectra without Fe^2+^ exposure) are shown in Figure 3. MetHb levels were 0.5 ± 0.5% (0.025 ± 0.025 *µ*M) for *t*_inc_=1 hour, 1.5 ± 0.5% (0.075 ± 0.025 *µ*M) for *t*_inc_=5 hours, and 5 ± 1% (0.25 ± 0.05 *µ*M) for *t*_inc_=24 hours, while the levels of hemolysis were 0.5 ± 0.5%, 1.0 ± 0.5%, and 10 ± 1%, respectively (Figures 3(a)–3(c)). After the influence of Fe^2+^1700 *μ*M on pRBC suspension, the MetHb level and hemolysis did not differ from the control values for *t*_inc_=1 hour, almost increasing to 20 ± 2% (1.0 ± 0.1 *µ*M) for *t*_inc_=5 hours and up to 82 ± 3% (4.10 ± 0.15 *µ*M) for *t*_inc_=24 hours, while the levels of hemolysis were 21 ± 3% and 58 ± 5%, respectively. A close correlation was established between the mean values of the MetHb level and the level of hemolysis, *r*_MetHb−Hemolysis_=0.98.

**Figure 4:**
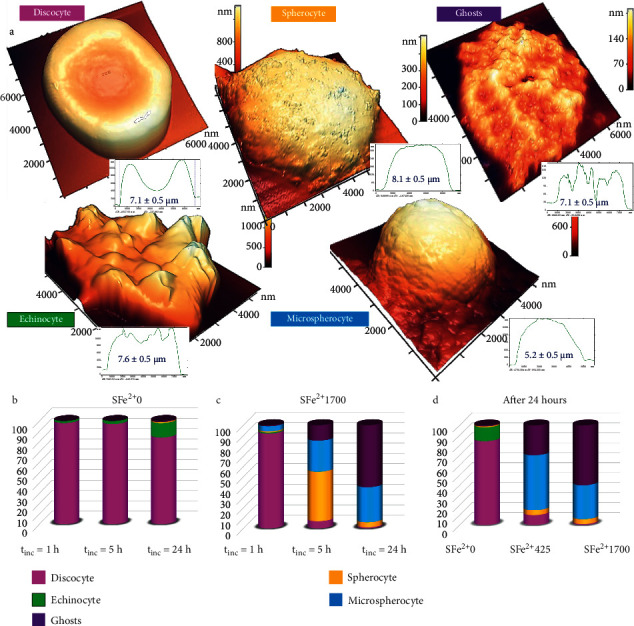
Typical forms of pRBCs after free Fe^2+^ exposure. (a) 3D AFM images of typical cell shapes after exposure of pRBCs to free Fe^2+^; color scales of cell heights are shown. On the right of each cell, the profile and its size are shown as the mean ± SD. (b, c) Histograms of the dynamics of changes in the percentage of each cell form for SFe^2+^ 0 and SFe^2+^ 1700, *t*_inc_ = 1, 5, and 24 (h). On the right is the color coding for the different cell shapes. (d) Histogram of the dynamics of changes in the percentage of each cell form after 24 hours for different concentrations of free iron. On the right is the color coding for the different cell shapes.

The formation of MetHb was accompanied by a significant change in cell forms (Figures 3 and 4). In the control sample, two types of cells were observed (Figure 4(b)): discocytes and echinocytes (approximately 3% at *t*_inc_=5 hours and approximately 14% at *t*_inc_=24 hours). After exposure to Fe^2+^1700 *μ*M at *t*_inc_=1 hour, the morphology of the cells changed insignificantly, and after *t*_inc_=5 hours, significant changes had already occurred. Four types of cells were observed (Figure 4(c)): 76 ± 4% were spherocytes and microspherocytes (approximately half), 18 ± 4% became ghosts, and only 6 ± 1% remained discocytes. After *t*_inc_=24 hours, 60 ± 4% of the cells became ghosts and 34 ± 2% were microspherocytes. There were small number of discocytes (1%) and spherocytes (5%).

Thus, the most part of the cells hemolyzed and became ghosts. We experimentally established that pRBC damage in result of Fe^2+^ exposure occurs through two mechanisms: (1) swelling of cells, the formation of spherocytes, and ultimately their hemolysis and formation of ghosts and (2) the formation of microspherocytes. These forms are irreversible and their formation, even in small amounts, can significantly reduce the quality of pRBCs and their functional ability. The effects of altering the pRBC morphology were enhanced with increasing concentrations of free Fe^2+^ in suspension (Figure 4(d)). An inverse correlation was established between the mean MetHb level and the mean number of discocytes on a smear *r*_MetHb−Discocyte_=−0.72.

We identified pRBC ghosts in AFM images, and accordingly, decreases in the level of optical density in the optical spectra were fixed (Figure 4). There was a good correlation of the percentage of ghosts on AFM images with the number of hemolyzed cells measured by the residual level in the absorption spectrum of the pRBC suspension: ghosts 18 ± 4% and hemolysis level 21 ± 3% after 5 hours of incubation, and ghosts 60 ± 4% and hemolysis level 58 ± 5% after 24 hours of incubation; thus, *r*_Hemolysis−Ghost_=0.99.

### 3.4. Bridges, Chains, and Clots of pRBCs Were Formed

**Figure 5:**
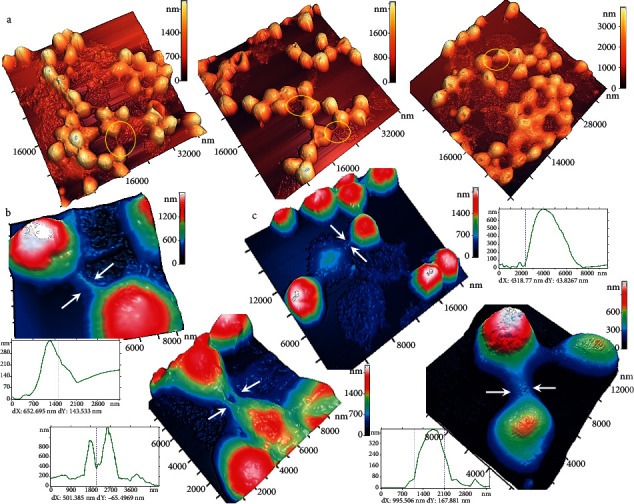
Formation of bridges, chains, and clots of pRBCs. (a) 3D AFM images of pRBCs after free Fe^2 +^ exposure. A color scale of heights is given for all images. Yellow circles show the areas of formation of bridges and chains. (b) 3D AFM images of typical bridges between cells and their profiles. A color scale of heights is given for all images. White arrows indicate the boundaries of the profiles. (c) Formation of a chain of two microspherocytes, between which there is a spherocyte in the process of hemolysis. Color scales of heights are shown. White arrows indicate the boundaries of the reduced profile.

In experiments, there were the self-organization of pRBCs. Connections between cells arose in the form of bridges, and chains were formed; as a result, they were combined into conglomerates and clots (Figure 5(a)). The height of the bridges between the cells ranged from 300 to 600 nm, the width ranged from 400 to 700 nm, and the length ranged from 20 to 200 nm. Some examples of bridges and chains are shown in Figure 5(b). Figure 5(c) shows the moment of chain formation from two microspherocytes, between which there is a spherocyte in the process of hemolysis, and bridges are formed between these three cells. Up to 10–12 cells were observed in chains. 30–40 cells formed clots. We observed these phenomena precisely after Fe^2+^ exposure for *t*_inc_=24 hours. The bridges were absent in the control and were practically not observed for *t*_inc_=60 min . The formation of such structures by free iron may increase the probability of blood clot forming in blood vessels.

The structure of aggregates in such clots was different from that of aggregates in the form of coin columns from control cells. We have already observed similar aggregate clots in the case of exposure of blood to carbon monoxide [53, 60] as well as zinc ions [61]. Perhaps, this effect is associated with a change in the ratio of hemoglobin derivatives or with partial local hemolysis. In the scientific literature, two alternative mechanisms of RBC aggregation have been conceived, namely a bridging model [62] and a local osmotic gradient model [63]. Based on the bridge model, large macromolecules are adsorbed on the cell surface and thereby connect two adjacent cells. When these bridges exceed disaggregation forces such as electrostatic repulsion, membrane deformation and mechanical shear, aggregation occurs [64]. These mechanisms may manifest themselves in the case of the action of excess iron on RBCs. Like agglutination, the phenomenon we observed is dangerous due to an increase in the formation of blood clots in vessels of various diameters.

### 3.5. The pRBC Membrane Stiffness Was Increased

**Figure 6:**
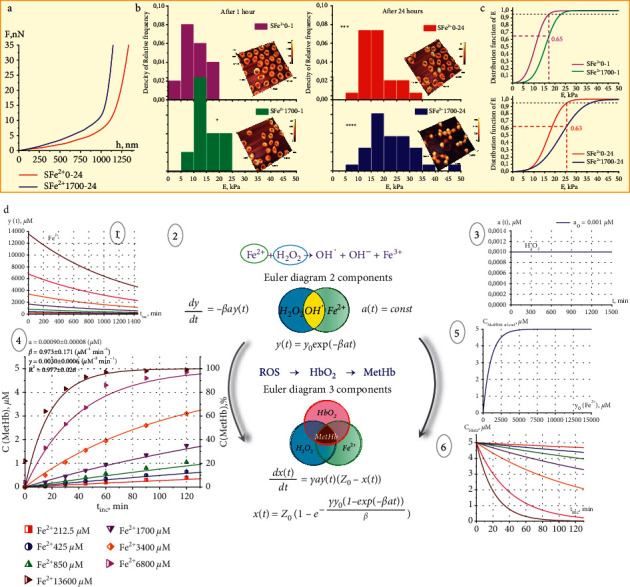
Increasing the pRBC membrane stiffness and a kinetic model. (a) Typical force curves for the control (SFe^2+^ 0–24) and experimental samples (SFe^2+^ 1700–24). (b) Histograms of the relative frequency density for Young's moduli of native pRBC membranes, *n* = 100 points for each histogram. For each histogram, the corresponding AFM images of pRBCs are shown. The bottom scale is the same as the top one; from 0 to 0.1 with step 0.02. The one-way ANOVA test followed by the Tukey post hoc test was used: ^*∗*^*p* < 0.05,  ^*∗*^ ^*∗*^ ^*∗*^*p* < 0.001, and  ^*∗*^ ^*∗*^ ^*∗*^ ^*∗*^*p* < 0.0001 compared to SFe^2+^ 0–1. (c) Distribution functions *F*(*E*) for SFe^2+^ 0 and SFe^2+^ 1700 after 1 and 24 hours. The 0.95 level is shown as a black dashed line. (d) Kinetic model of changes in MetHb content. 1: graph *y*(*t*) during the interaction of Fe^2+^ ions with peroxide at constant concentration of H_2_O_2_; 2: model equations; 3: graph *a*(*t*) over time (quasi-steady state condition); 4: increase in the *C*_MetHb_(*t*) in the range of incubation time *t*_*inc*_ = 0–120 min (symbols indicate experimental data and lines correspond the fitting functions according to calculated parameters); fitting curves were calculated according model, equation (10); 5: the dependence of steady state level of MetHb content on initial concentration *С*_Fe^2+^_ : *C*_MetHb_(*С*_Fe^2+^_), equation (11); 6: graph of the *C*_HbO_2__(*t*) decrease.

In our experiments, morphological changes and the formation of MetHb were accompanied by an increase in pRBC membrane stiffness as a result of free Fe^2+^ exposure of the blood. AFS was used to investigate the deep bending of pRBC membranes up to *h* = 1000–1300 nm under the action of a probe (Figure 6(a)). Typical force curves for the control and experimental samples for *t*_inc_=24 hours are shown in Figure 6(a).

The force curve for the cell membrane after exposure to *F*_Fe^2+^1700_(*h*) is sharper than that for the control cell *F*_Fe^2+^0_(*h*) (Figure 6(a)). The 100 measured force curves were calculated using the Hertz formula, and histograms of Young's modulus for membranes of these groups were plotted (Figure 6(b)). The corresponding mean and variances were calculated: *E*_Fe^2+^0_=11 ± 4 kPa and *E*_Fe^2+^1700_=17 ± 5 kPa for *t*_inc_=1 h and *E*_Fe^2+^0_=15 ± 5 kPa and *E*_Fe^2+^1700_=23 ± 8 kPa for *t*_inc_=24 h. Figure 6(c) shows the distribution functions of Young's modulus for *t*_inc_=1 h and for *t*_inc_=24 h, corresponding to the study and control groups. Based on these curves, it was found that after *t*_inc_=1 h after exposure to Fe^2+^ 1700 *μ*M, 35% of pRBC membranes had Young's modulus values that were beyond the control values (at a level of 0.95). The results were almost the same after *t*_inc_=24 h, 37%. These results indicate that almost one-third of the cells had increased stiffness, which reduced their deformability and potentially caused microcirculation disorders. Earlier studies showed that the reason for the change in the stiffness of RBC membranes with an increase in oxidative processes is a change in the structure of the cytoskeleton of cells [46, 52, 58, 65–67].

### 3.6. Kinetic Model of Changes in Hemoglobin Derivative Concentrations

The biomarker for the development of oxidative processes in RBC suspensions under the influence of various physicochemical factors is the MetHb level [52, 54]. To describe the change in the MetHb level under the action of free iron, we proposed a mathematical model based on kinetic equations. The kinetic model is derived from fundamental principles as mass balance considering a constant volume and describes interactions between network components and the processes they undergo. A first-order ordinary differential equation (ODE) based on the law of mass action stating that the rate of the chemical reaction is directly proportional to the product of the reactants allowed us to describe the kinetics of the processes.

#### 3.6.1. Model Assumptions

The following assumptions were made that define the model's validity range:

(1) The initial main triggering reaction for subsequent processes is the reaction of the interaction of iron ions Fe^2+^ with ROS, namely, with peroxide H_2_O_2_:
  (3) $$\begin{array}{c} {\text{Fe}}^{2+}+{\text{H}}_{2}{\text{O}}_{2}⟶{\text{OH}}^{\cdot }\;+{\text{OH}}^{-}+{\text{Fe}}^{3+} \end{array}$$
[9, 31, 47, 48]. During the subsequent redox reactions, the conversion of HbO_2_ to MetHb occurs.
(2) We consider steady-state condition with respect to *С*_H_2_O_2__.The concentration of H_2_O_2_ in the system is maintained constant, *С*_H_2_O_2__=const. The concentrations of peroxide in the blood and, accordingly, in the working solution are very low. However, it should be taken into account that in the course of subsequent ROS reactions, H_2_O_2_ is formed again [68, 69].

#### 3.6.2. Two-Component Kinetic Model: The Interaction of Free Fe^2+^ with H_2_O_2_

*Derivation of a Differential Equation and its Solution*. We suggest the kinetic model describing the interaction of free Fe^2+^ with H_2_O_2_ in (equation (3)).

Let us denote

*y*(*t*)=*С*_Fe^2+^_(*t*); (d*y*/d*t*) is the rate of change in the concentration of iron ions Fe^2+^; *β* is the rate constant; and *a*=*С*_H_2_O_2__=const.

We rely on the fact that the rate of the chemical reaction (equation (3)) is directly proportional to the product of the reactant concentrations, and then the ODE is as follows:

(4) $$\begin{array}{c} \frac{\text{d}y}{\text{d}t}=-\beta ay\left(t\right). \end{array}$$

Initial condition is

(5) $$\begin{array}{c} \text{at }t=0,\\ y=\;y_{0}. \end{array}$$

Solving the differential equation (equation (4)) by the method of separation of variables and taking into account the initial conditions (equation (5)), we get the dependence of the Fe^2+^ change on time:

(6) $$\begin{array}{c} y\left(t\right)=y_{0}\exp\left(-\beta at\right). \end{array}$$

Figure 6(d), 1 shows the graphs of the decrease in the concentration of iron ions *y*(*t*) during their interaction with peroxide for a constant concentration *С*_H_2_O_2__=1 nM (Figure 6(d), 3), which was in our experiments.

For illustration, an Euler diagram for the two-component reaction (green, Fe^2+^ ions and blue, H_2_O_2_ molecules) is presented in Figure 6(d), 2. The intersection area is highlighted in yellow and corresponds to the reaction product hydroxyl radical OH^·^.

#### 3.6.3. Three-Component Model: Interaction of Free Fe^2+^, H_2_O_2_, and HbO_2_ in the Lysate

*Derivation of a Differential Equation and Its Solution.*H_2_O_2_ is a long-lived representative of ROS that can diffuse over long distances. However, at the same time, it has a lower reactivity in comparison with the rest of the ROS which are short-lived and diffuse over short distances but exhibit high reactivity. As a result of the reaction (equation (3)), a hydroxyl radical OH^·^ is formed, which is highly reactive and short-lived. It is the hydroxyl radical that triggers and participates in subsequent redox reactions. The radical OH^·^ will be able to enter into further reactions with other closely located ROS and HbO_2_.

Let us denote

(7) $$\begin{array}{c} x\left(t\right)=\;C_{\text{MetHb}}\left(t\right). \end{array}$$

*Z*_0_ is the initial concentration of HbO_2_; under the experimental conditions *Z*_0_=5 ± 0.5 *μ*M and (*Z*_0_ − *x*(*t*)) is the concentration of HbO_2_ at any time.

According to the mass action law, the rate of the chemical reaction is directly proportional to the product of the reactant concentrations:

(8a) $$\begin{array}{c} \frac{\text{d}x\left(t\right)}{\text{d}t}=\gamma ay\left(t\right)\left(Z_{0}-x\left(t\right)\right), \end{array}$$

where *γ* is the rate constant.

Initial condition: we assume that the initial level of MetHb at *t* = 0 is

(8b) $$\begin{array}{c} x_{0}=0. \end{array}$$

Taking into account the dependence equation (6), we write down the ODE (8a) in the form

(9a) $$\begin{array}{c} \frac{\text{d}x\left(t\right)}{\text{d}t}=\gamma ay_{0}\left(Z_{0}-x\left(t\right)\right)\text{exp}\left(-\beta at\right). \end{array}$$

This is the first-order ODE. It is solved by the method of separation of variables:

(9b) $$\begin{array}{c} \int \frac{\text{d}x}{Z_{0}-x}=\int \gamma ay_{0}\exp\left(-\beta at\right)\text{dt}, \end{array}$$

(9c) $$\begin{array}{c} \ln\left(Z_{0}-x\right)=-\frac{\gamma y_{0}\;\exp\left(-\beta at\right)}{\beta }+B, \end{array}$$

where B is the arbitrary constant.

Taking into account the initial condition equation (8b), we can find that

(9d) $$\begin{array}{c} B=\ln Z_{0}+\frac{\gamma y_{0}}{\beta }. \end{array}$$

Substituting equation (9d) into equation (9c), we get the dependence of MetHb concentration change:

(10) $$\begin{array}{c} x\left(t\right)=Z_{0}\left(1-e^{-\left(\gamma y_{0}\left(1-\exp\left(-\beta at\right)\right)/\beta \right)\;}\;\right). \end{array}$$

The steady state level of MetHb can be found depending on the initial concentration of Fe^2+^ ions(*y*_0_).

In this case, in equation (10), *t*⟶*∞*. Then,

(11) $$\begin{array}{c} x{\left(t\right)}_{\text{stst level}}=Z_{0}\left(1-e^{-\left(\gamma y_{0\;}/\beta \right)\;}\right). \end{array}$$

The units of reactant concentrations are *Z*_0_(*μM*), *x*(*μM*), *y*_0_(*μM*),  and *a*(*μM*); the units of parameters are *β*(*μM*^−1^min^−1^) and *γ*(*μM*^−2^min^−1^); and the time is *t* (min).

For illustration, an Euler diagram for the 3-component reaction (red: HbO_2_ molecules) is presented in Figure 6, 2. The intersection area is highlighted in brown and corresponds to the final reaction product MetHb.

#### 3.6.4. Fitting of Experimental Data

Nonlinear curve fitting of the experimental data of the MetHb concentration change (Figures 2(b) and 2(e)) was performed according to equation (10) by using Origin Pro 2019. The unknown values of concentration *a* and rate constants *β* and *γ* were considered as model parameters which describe the experimental data in the best way. The known values were *x*(*t*) and *y*_0_. The criterion for the optimal fitting was that the theoretical values of *x*_theor_(*t*) fit best the experimental data *x*_exper_(*t*), at a level *R*^2^ > 0.96. As a result of the fitting, the values of *a*, *β*, and *γ* were obtained for each curve corresponding to a different initial iron level *y*_0_. Taking into account the data averaging, we obtained that *a* = 0.00090 ± 0.00008 (*μM*), *β* = 0.973 ± 0.171 (*μM*^−1^min^−1^), *γ* = 0.0030 ± 0.0006 (*μM*^−2^min^−1^), and *R*^2^ = 0.977 ± 0.026.

It is noteworthy that the theoretically found concentration of *a*(*C*_H_2_O_2__), which was in the equations as quasi-steady state condition, coincided with the experimental value with an accuracy of 5–10%. This also indicates the adequacy of the model and, accordingly, the mechanism of the kinetics of the interaction of H_2_O_2_, Fe^2+^, and HbO_2_.

Figure 6(d), 4 shows lines according to dependence (equation (10)) for different initial concentrations of Fe^2+^ ions which fit the experimental data (indicated by symbols) from Figure 2(d) in the first *t*_*inc*_ = 120 min. The steady state level of the MetHb content (saturation) and the time to reach it (or the rate) depend on the initial concentration of Fe^2+^ according to equation (11) (Figure 6(d), 5). With an increment in the initial *C*_Fe^2+^_, the steady state level of MetHb increases in good agreement with the experimental data (Figure 2(d)). This is the confirmation of the adequacy of the model.

The increase in MetHb was accompanied by a decrease in HbO_2_ (Figure 6(d), 6). At a high initial concentration of *C*_Fe^2+^_ = 6800–13600 *μ*M, the rate of the Fenton reaction and, accordingly, the rate of decrease in HbO_2_ will be large, and accordingly, for a short incubation time of 100–200 min, almost all HbO_2_ will be converted to MetHb (Figure 6(d), 6). The reason for the steady state of the MetHb level in this case is the use of all HbO_2_ molecules during this time of observation. In another situation, with a low initial concentration of iron, the rate of MetHb formation will be insignificant. In this case, a steady state of MetHb concentration will be observed but already at a lower level. The reason for the steady state will be already an insufficient amount of iron ions decreasing with the time.

### 3.7. RBCs as a Key Link in the Influence of Free Iron Overload: The Scheme Representation

Despite the publication of numerous studies concerning the problems of iron overload, the comprehensive study of excess iron influence on RBCs remains limited. To better identify the processes in RBCs under iron overload, we used biophysical methods to perform a modeling study *in vitro*.

**Figure 7:**
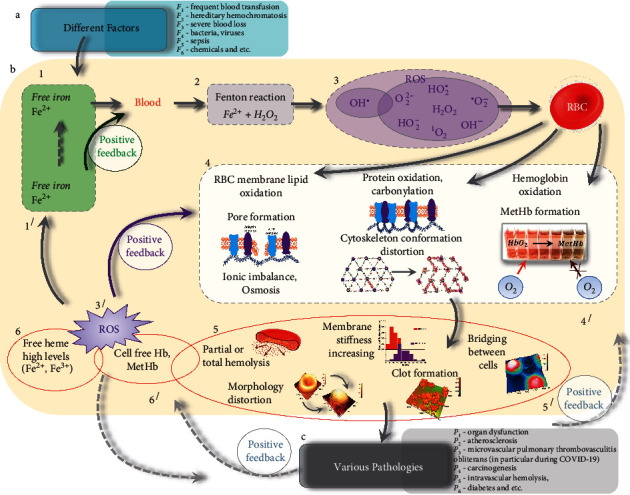
The scheme of the mechanism of the toxic action of excess iron on RBCs, possible distortions in constituent elements of cells, and possible pathophysiological consequences in the clinic. (a) Various factors *F*_*i*_, which can serve as a source of free iron. (b) Violations in RBCs. 1, 1^/^: formation of free iron; 2: fenton reaction. 3, 3^/^: ROS formation; 4, 4^/^: violations in constituent elements of RBCs; 5, 5^/^: pathological processes in RBCs; 6, 6^/^: formation of cell-free Hb, free heme. (c) Various clinical consequences *P*_*i*_. There are indicated positive feedbacks in the process development.

In a healthy organism, there is a dynamic balance of concentrations of Fe^2+^/Fe^3+^, H_2_O_2_ /ROS, and HbO_2_/MetHb. Normally, the iron balance is maintained by iron transport proteins [1]. Free iron overload occurs in a number of diseases (Figure 7 (a)). In critically ill patients, particularly in cases of sepsis, the capacity to detoxify cell-free hemoglobin is reduced [16, 18]. As a result, the equilibrium is disturbed, and the system goes into another state, while collective disproportions arise in all elements of the system in one way or another (Figure 7 (b)).

In this chain of pathological process development, RBCs play a key role (Figure 7 (b) 1–6). Excess iron accumulation causes distortions through the production of ROS [2] via the Fenton reaction (Figure 7 (b1) and (b2)), with the generation of toxic hydroxyl OH^·^ radicals, which are highly reactive and toxic [70]. The generation of ROS (Figure 7 (b3)) leads to the peroxidation of membrane lipids and cellular proteins, resulting in membrane and cytoskeleton distortions and hemoglobin derivative transformations (Figure 7 (b4)). As has already been noted repeatedly, RBC hemolysis is a dangerous consequence of the action of free iron on the blood. When OH^•^ interacts with membrane lipids, chain reactions of lipid peroxidation occur (Figure 7 (b4)). As a result, the hydrophilicity of bonds in lipid tails increases locally, and local hydraulic pores are formed. This leads to disruption of the ionic balance in RBCs, and osmosis occurs. This leads to a change in cell morphology as well as partial and complete hemolysis of spherocytes.

Also, high iron condition might induce RBC surface protein denaturation, leading to their adhesion to each other. Probably with excess iron, changes in the band 3 proteins and spectrin occur (Figure 7 (b4)). This leads to change of cytoskeleton and membrane surface nanostructure. We observed similar phenomena using AFM studies of the influence of Zn ions, UV radiation, and hemin, during long-term storage of pRBCs [52, 57, 71, 72]. Clusterization of protein may be the reason of bridge and clot formation.

Serious disorders in the pRBCs due to ROS in result of iron overload are shown in Figure 7 b4 and b5, and we established those in our experiments *in vitro* (Figures 3 and 4).

The effect of Fe^2+^ on RBCs occurs under conditions positive feedback in the system *free iron–blood* (Figure 7). An excess of free iron leads to the appearance of free HbO_2_, MetHb, and to a high concentration of free hemes, which in turn leads to free iron and ROS [9, 73]. These positive feedbacks are shown in Figure 7 c, b 1^/^, 3^/^, 4^/^, 5^/^, and 6^/^.

Disorders in RBCs can cause abnormalities cascade of pathological processes in organisms (Figure 7 (c)) [26, 27, 37, 73–83].

RBCs may be potential sources of clinical changes *in vivo* in the cardiovascular system (Figure 7 (c)). In particular, the clinical consequence of excess iron is atherosclerosis, which increases the possibility of hemolysis of pRBCs, therefore leading to an increase in the production of free iron and oxidized products. In addition to hypoperfusion, up to a complete blockage of blood flow, massive hemolysis occurs with the appearance of free hemoglobin and the increase in the level of free iron. A vicious cycle is created when iron initiates an inflammatory process, including thrombovasculitis, and the blockade of micro- and macrocirculation creates conditions for hemolysis and an increase in the level of free heme iron in the blood serum [8, 26, 84]. As a result, a rapid increase in respiratory insufficiency occurs due to blockage of the pulmonary blood flow. Microvascular pulmonary thrombovasculitis obliterans, which is characterized by thrombosis of not only microcirculatory vessels but also larger vessels, is typical for COVID-19.

In our *in vitro* studies, we tried to answer the question what disorders can occur in RBCs which can modify their functions. We established the pattern of the simultaneous development of heterogeneous processes under the action of excess iron: changes in the morphology of pRBCs, the formation of bridges between cells, the formation of clots (such as pRBC agglutination), the formation of MetHb, and increasing of membrane Young's modulus (Figures 2–7 (b5), (b6)).

## 4. Conclusion

In conclusion, iron is useful and nontoxic, when bound in our bodies in optimal quantities. However, overload of free iron is dangerous and can cause powerful oxidative stress. In biophysical experiments *in vitro*, we have experimentally established a complex of changes that can occur in pRBCs and lead to the development of pathological processes. Thus, we have shown that pRBCs may be the key link in the pathology influence of free iron overload. Patterns established in our experiments *in vitro* can allow to expand the understanding of the mechanisms of the toxic adverse effect of excess free iron on the blood and the circulatory system, and, consequently, to develop adequate methods for patient treatment.

## Data Availability

The data used to support the findings of this study are available from the corresponding author upon request.
